# Controlled tuning of HOMO and LUMO levels in supramolecular nano-Saturn complexes†

**DOI:** 10.1039/d4ra07068b

**Published:** 2024-12-12

**Authors:** Maria Maqbool, Khurshid Ayub

**Affiliations:** a Department of Chemistry, COMSATS University Abbottabad Campus KPK 22060 Pakistan khurshid@cuiatd.edu.pk +92-992-383591

## Abstract

Optoelectronics usually deals with the fabrication of devices that can interconvert light and electrical energy using semiconductors. The modification of electronic properties is crucial in the field of optoelectronics. The tuning of the highest occupied molecular orbital (HOMO) and lowest unoccupied molecular orbital (LUMO) and their energy gaps is of paramount interest in this domain. Herein, three nano-Saturn supramolecular complex systems are designed, *i.e.*, Al_12_N_12_@S-belt, Mg_12_O_12_@S-belt, and B_12_P_12_@S-belt, using S-belt as the host and Al_12_N_12_, Mg_12_O_12_, and B_12_P_12_ nanocages as guests. The high interaction energies ranging from −22.03 to −63.64 kcal mol^−1^ for the complexes demonstrate the stability of these host–guest complexes. Frontier molecular orbital (FMO) analysis shows that the HOMO of the complexes originates from the HOMO of the host, and the LUMO of the complexes originate entirely from the LUMO of the guests. The partial density of states (PDOS) analysis is in corroboration with FMO, which provides graphical illustration of the origin of HOMO and LUMO levels and the energy gaps. The shift in the electron density upon complexation is demonstrated by the natural bond orbital (NBO) charge analysis. For the Al_12_N_12_@S-belt and B_12_P_12_@S-belt complexes, the direction of electron density shift is towards the guest species, as indicated by the overall negative charge on encapsulated Al_12_N_12_ and B_12_P_12_. For the Mg_12_O_12_@S-belt complex, the overall NBO charge is positive, elaborating the direction of overall shift of electronic density towards the S-belt. Electron density difference (EDD) analysis verifies and corroborates with these findings. Noncovalent interaction index (NCI) and quantum theory of atoms in molecules (QTAIM) analyses signify that the complexes are stabilized *via* van der Waals interactions. Absorption analysis explains that all the complexes absorb in the ultraviolet (UV) region. Overall, this study explains the formation of stable host–guest supramolecular nano-Saturn complexes along with the controlled tuning of HOMO and LUMO levels over the host and guests, respectively.

## Abstract 2

Controlled tuning of HOMO and LUMO levels.

## Graphical abstract

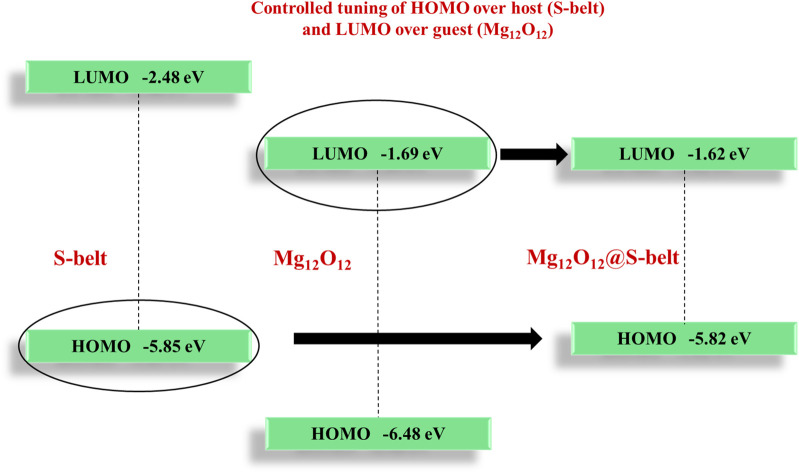

## 1. Introduction

Two-dimensional materials, such as graphene, are a major focus in materials science due to their novel optoelectronic properties. At room temperature, graphene exhibits zero band gap (energy gap) along with higher carrier mobility.^1^ To date, various strategies have been designed to induce energy gaps in zero energy gap materials. Similarly, different methods are adopted to decrease the energy gaps of the materials with greater energy gaps. Some of the methods for altering the energy gap include doping,^2–4^ strain engineering,^5–8^ van der Waals heterostructures,^9–11^ lateral confinement,^12,13^ and adsorption of atoms.^14–17^ The purpose behind the tuning of energy gaps is to improve the semiconducting characteristics of the systems. The optical properties of GaN alloys can be controlled by tuning their energy gaps.^18^ Due to the excellent electrical conductivity and favorable photovoltaic properties, fullerene-based molecules have attracted considerable attention in the last two decades.^19^

Doping is one of the extensively used methods to enhance the conductivity of materials and improve their optoelectronic characteristics. Tayyab *et al.* investigated the effect of Mg and Be doping on graphene. The findings indicated the considerable modification in electronic and structural properties after doping compared to bare graphene. They concluded that the doped graphene can be used for optoelectronic devices due to reasonable energy gap.^20^ Strain engineering is proved to significantly reduce the energy gap of the materials. Specifically, the vertical strain impacts the gap size, resulting in a transition from semiconductor to metal.^21,22^ In van der Waals (vdW) heterostructures, different transition metal dichalcogenide (TMD) layers are vertically stacked. A key characteristic that influences the functionality of vdW heterostructures is the alignment of the valence band maxima (VBM) and conduction band minima (CBM) of adjacent layers. To create a functional vdW heterostructure with required characteristics, one approach is to explore the available 2D materials for combinations of layers where the band gap sizes and band alignment meet the desired requirements. However, this process can be time-consuming and may involve materials that are challenging to acquire or work with.^23^ Moreover, external pressure and external electric field also play important roles in energy gap engineering, but some drawbacks are also associated with these methods. Energy gap reduction is achievable through all the previously mentioned approaches, but the fine-tuning of HOMO and LUMO energy levels over the distinct species cannot be achieved. A viable solution to the challenge of energy level tuning is the design of host–guest chemistry.

Nano-Saturn complex system is one of the examples of host–guest supramolecular assembly. A nano-Saturn system is comprised of spherical nanocages as the guest species and annular macrocyclic belts as the host species. The nano-Saturn host–guest complexes are stabilized *via* van der Waals interactions. Hydrocarbon nanobelts and heteroatom-doped hydrocarbon nanobelts are commonly used as hosts in such systems. The concave cavity inside the carbon nanobelts can act as a host for the convex structures, *e.g.*, fullerenes. Cycloparaphenylenes (CPPs) and their derivatives can also be used as host molecules for the encapsulation of guests in host–guest chemistry. The derivatives of cycloparaphenylenes show very small HOMO–LUMO energy gaps due to their donor–acceptor properties. In order to tune the electronic characteristics of CPPs, various electron-donating groups have been substituted.^24^ Nitrogen atom is extensively used for doping in carbon nanomaterials. Due to the presence of an extra electron in N, compared to C, the carbon atoms adjacent to N in carbon nanomaterials have greater positive charge density. The N atoms also give electrons to the sp^2^ carbon skeleton, thus enhancing the electronic properties (such as electrical conductivity) of these nanomaterials. Recently, sulphur and oxygen have gained attention as the dopants in carbon nanomaterials due to their ability to enhance the electrical properties, especially electrical conductivity and capacitance.^25^ Shi *et al.* investigated that the structural features and capability of macrocyclic hosts (belts) to recognize other molecules (guests) was governed by the constituent building blocks and the way these blocks were interconnected.^26^ Regarding this, carbon, heteroatom-doped nanobelts and their complexes with C_60_ were studied by George *et al.* They thoroughly examined the characteristics of the ground as well as the excited states of these complexes, which enabled the establishment of correlations between their structures and properties.^27^

The most common spherical nanocages in host–guest systems are fullerenes. Fullerenes consist of sp^2^ hybridized carbon atoms that are organized in the form of spherical nanocages.^28^ They are comprised of pentagonal and hexagonal rings. The unique chemical, mechanical and physical properties of fullerenes are due to their zero-dimensional spherical nanostructures. These properties include higher tensile strength, efficient electrical and thermal conductivity, greater surface area and good electron donor and acceptor abilities. Fullerenes have found applications in solar cells, medicine, gas storage, fuel cells, fullerene switches, superconductors, chemical sensors, semiconductors and optoelectronics.^29,30^ Other fullerene-like nanostructures such as Al_12_P_12_, Al_12_N_12_, B_12_N_12_, Be_12_O_12_, Mg_12_O_12_, Si_12_C_12_, Ca_12_O_12_, and B_12_P_12_ are also used in optoelectronic and photocatalytic applications due to their structural, optical and electronic properties.^31^

There are several factors that can play a role in controlling the host–guest chemistry, both in experimental and theoretical chemistry. The major factor controlling the host–guest chemistry is the host–guest size compatibility.^32–36^ The literature highlights how host–guest systems at liquid–solid interfaces are controlled by cavity properties, surface interactions, and environmental conditions. The findings reveal that HG encapsulation is influenced by factors such as host cavity dimensions, molecular flexibility, electronic interactions (π–π stacking, donor–acceptor behavior, and dipole effects), and substrate confinement. Notably, C_60_ preferentially occupies the rim of macrocyclic hosts due to donor–acceptor interactions, and surface-induced effects lead to unique stoichiometries such as 1 : 1.^33^

In this study of host–guest supramolecular chemistry, three nano-Saturn type complexes are designed for the fine-tuning of HOMO and LUMO energy levels over the host and guest species, respectively. The fullerene-like nanocages (*i.e.*, Al_12_N_12_, Mg_12_O_12_, and B_12_P_12_) are selected as guests and S-belt (composed of seven 1,4-benzothiazine units) as the host species. The suitable alignment of the HOMO and LUMO levels of these species make them ideal candidates for this study. The HOMO of the host (S-belt) is above the HOMO of the guests (Al_12_N_12_, Mg_12_O_12_, and B_12_P_12_) whereas the LUMO of guests is below that of the host. Thus, it is predicted that after the nano-Saturn complex formation, the HOMO and LUMO levels of the complex will lie closer to the host and guests, respectively, resulting in the tuning of HOMO and LUMO levels over the distinct species.

## 2. Methodology

Gaussian 09 (ref. 37) was used to perform all the density functional theory (DFT) calculations. To build and visualize the structures, GaussView 5.0 ^38^ was used. The DFT functional, *i.e.*, ωB97XD and pople-type double-ζ basis set along with polarization function, *i.e.*, 6-31G(d,p), are used for geometry optimization.^39^ The frequency calculation using the same functional and basis set shows that all the frequencies are real, demonstrating true minimum nature of stationery points on potential energy surfaces. For quantum theory of atoms in molecules (QTAIM) and noncovalent interaction index (NCI) analyses at the same functional and basis set, the Multiwfn^40^ and visual molecular dynamics (VMD)^41^ software packages are used. ωB97XD is a range-separated hybrid functional (RSH) with 22.2–100% Hartree–Fock exchange.^42^ The electronic properties, *i.e.*, the energy of HOMO, LUMO and *E*_gap_, were calculated using another DFT functional, *i.e.*, B3LYP, accompanied by the same basis set (as used for geometry optimization *i.e.*, 6-31G(d,p)). B3LYP is a global hybrid functional (GH) with 20% Hartree–Fock exchange. B3LYP is a well-known method for the investigation of electronic properties.^43–47^ Hence, it is only used for the calculation of electronic properties, *i.e.*, natural bond orbital (NBO), electron density difference (EDD), frontier molecular orbital (FMO) and density of states (DOS) analyses. Moreover, the ωB97XD functional has dispersion correction factor, due to which it is more suitable for the evaluation of interaction energies (*E*_int_).^36^ For the calculation of *E*_int_ of the complexes, the following equation is used.

1 *E*_int_ = *E*_complex_ − (*E*_S-belt_ + *E*_nanocage_)

In eqn (1), *E*_int_ is the resulting interaction energy, while *E*_complex_, *E*_S-belt_ and *E*_nanocage_ are the energies of complexes, S-belt and nanocages (Al_12_N_12_, Mg_12_O_12_, and B_12_P_12_), respectively. Moreover, time-dependent density functional theory calculations (*i.e.*, TD-DFT) at the ωB97XD/6-31G(d,p) level are employed for absorption analyses of the individual components (host–guest species) as well as complexes.

## 3. Results and discussion

### 3.1. Geometry optimization and interaction energy calculation

**Fig. 1:**
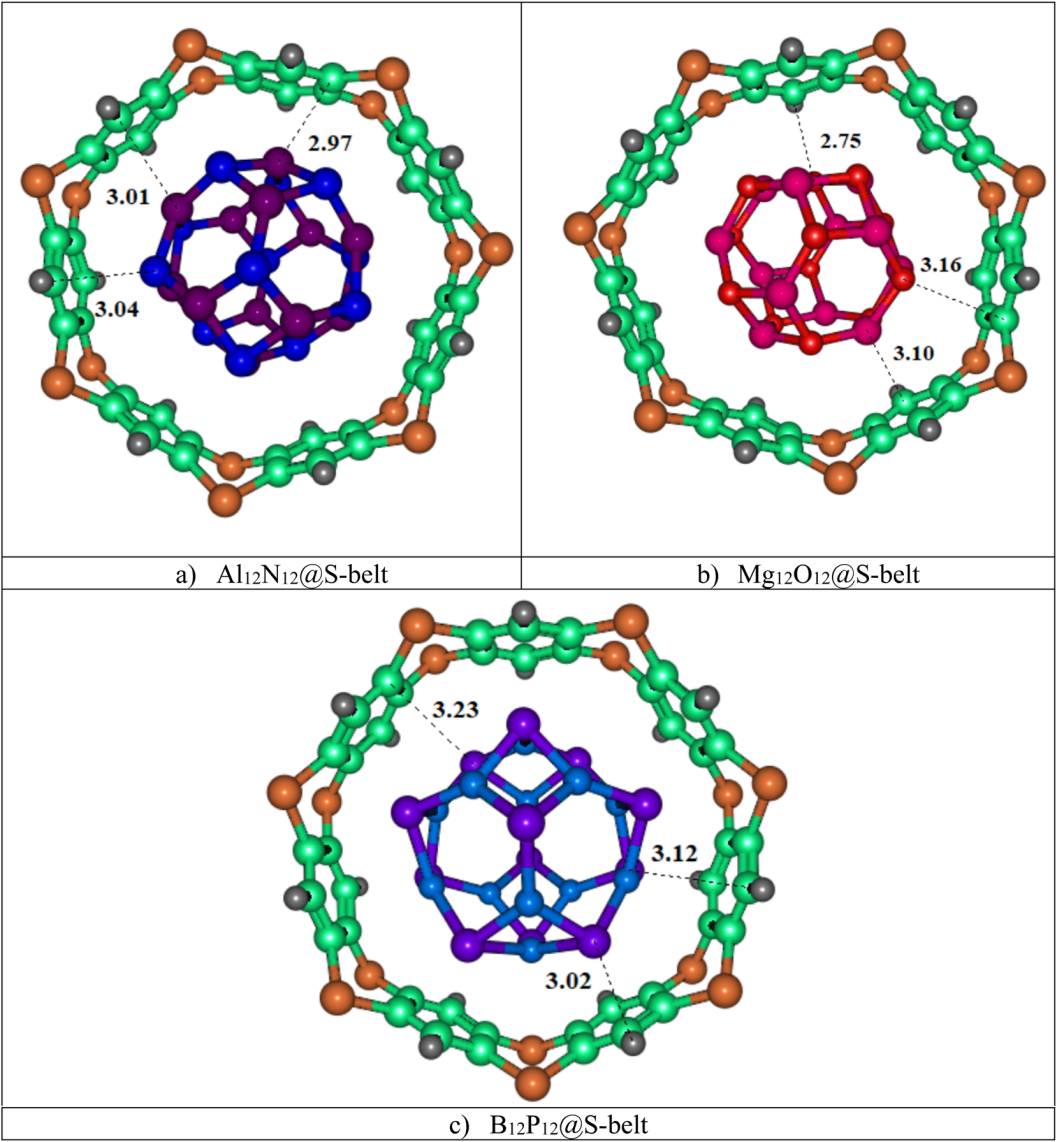
Stable optimized geometries of the (a) Al_12_N_12_@S-belt, (b) Mg_12_O_12_@S-belt, and (c) B_12_P_12_@S-belt complexes.

In this study, three host–guest supramolecular complexes (Al_12_N_12_@S-belt, Mg_12_O_12_@S-belt, and B_12_P_12_@S-belt) were assembled. The host used in the study is the S-belt made of seven 1,4-benzothiazine units. The cavity inside the S-belt is sufficient for the encapsulation of the guest species. The guests are three fullerene-like nanocages, also called heterofullerenes, or non-carbon fullerenes (inorganic fullerenes), *i.e.*, Al_12_N_12_, Mg_12_O_12_, and B_12_P_12_. Al_12_N_12_ and B_12_P_12_ are fullerene-like nanostructures of groups III–V, whereas Mg_12_O_12_ is the fullerene-like nanostructure of groups II–VI. The optimized structures of the designed host–guest complexes are reported in Fig. 1. Likewise, Table 1 shows the interaction energies (*E*_int_) and interaction distances (*D*_int_) of the optimized host–guest complexes. The Al_12_N_12_@S-belt complex shows *D*_int_ ranging from 2.97 to 3.04 Å, *i.e.*, lesser *D*_int_ compared to that of the other complexes. Consequently, the *E*_int_ of the complex is greater compared to that of the other two complexes, *i.e.*, −63.64 kcal mol^−1^. Similarly, the *D*_int_ (interaction distance between the nanocage and belt) for the Mg_12_O_12_ range of 2.75–3.16 Å, and *E*_int_ for the same complex is −58.02 kcal mol^−1^. The *D*_int_ for B_12_P_12_ is greater compared to the former two complexes, resulting in a lesser *E*_int_, *i.e.*, −22.03 kcal mol^−1^. The trends observed in *E*_int_ and *D*_int_ for the complexes are Al_12_N_12_@S-belt > Mg_12_O_12_@S-belt > B_12_P_12_@S-belt, and Al_12_N_12_@S-belt < Mg_12_O_12_@S-belt < B_12_P_12_@S-belt. The trend observed in the values of *E*_int_ can be attributed to the greater stability of the guest species inside the cavity of the host in the former two complexes compared to the latter one. Overall, all the complexes have reasonable *E*_int_, verifying the stability of the designed supramolecular complexes.

**Table 1:** Interaction energies and interaction distances of the Al_12_N_12_@S-belt, Mg_12_O_12_@S-belt, and B_12_P_12_@S-belt complexes

Complexes	*A*_ad_ (fullerene-belt)	*D*_int_ (Å)	*E*_int_ (kcal mol^−1^)
Al_12_N_12_@S-belt	Al1⋯C4	2.97	−63.64
	Al2⋯N5	3.01	
	N3⋯C6	3.04	
Mg_12_O_12_@S-belt	Mg1⋯C4	2.75	−58.02
	Mg2⋯C5	3.10	
	O3⋯C6	3.16	
B_12_P_12_@S-belt	P1⋯H4	3.02	−22.03
	P2⋯C5	3.23	
	P3⋯C6	3.12	

### 3.2. FMO analysis

The optoelectronic properties of the designed complexes are determined *via* frontier molecular orbital (FMO) analysis. The energies of HOMO, LUMO and *E*_gap_ for the bare host and guest as well as complexes are given in Table 2. The values of *E*_HOMO_, *E*_LUMO_ and *E*_gap_ for the bare S-belt are −5.85, −1.42 and 4.43 eV, respectively. For Al_12_N_12_, the values of *E*_HOMO_, *E*_LUMO_ and *E*_gap_ are −6.53, −2.48 and 4.04 eV, respectively. Similarly, the values of *E*_HOMO_, *E*_LUMO_ and *E*_gap_ for Mg_12_O_12_ are −6.84, −1.69, and 5.14 eV, respectively, and these values for B_12_P_12_ are −6.84, −2.78, and 33.02 eV, respectively.

**Table 2:** Energies of the HOMO, LUMO, and energy gaps of the host, guest species and the complexes (*i.e.*, Al_12_N_12_@S-belt, Mg_12_O_12_@S-belt, and B_12_P_12_@S-belt)

Compounds	*E*_HOMO_	*E*_LUMO_	*E*_gap_	NBO|*e*|
S-belt	−5.85	−1.42	4.43	—
Al_12_N_12_	−6.53	−2.48	4.04	—
Al_12_N_12_@S-belt	−5.80	−1.96	3.83	−0.307
Mg_12_O_12_	−6.84	−1.69	5.14	—
Mg_12_O_12_@S-belt	−5.82	−1.62	4.20	0.064
B_12_P_12_	−6.84	−3.10	3.74	—
B_12_P_12_@S-belt	−5.80	−2.78	3.02	−0.163

After complexation, the values of *E*_HOMO_, *E*_LUMO_ and *E*_gap_ for Al_12_N_12_@S-belt are −5.80, −1.96, and 3.83 eV, respectively. Likewise, these values for Mg_12_O_12_@S-belt are −5.82, −1.62 and 4.20 eV, respectively, whereas the corresponding values for the B_12_P_12_@S-belt complex are −5.80, −2.78, and 3.02 eV, respectively. Hence, after complex formation, it is observed that the energy of the HOMO of the complex closely resemble that of the bare host (*i.e.*, S-belt) and the energy of the LUMO of the complex is close to the energy of LUMO of the bare guests (*i.e.*, Al_12_N_12_, Mg_12_O_12_, and B_12_P_12_). Moreover, in comparison to the bare host and guests, the energy gaps of the complexes are reduced in all the three cases.

**Fig. 2:**
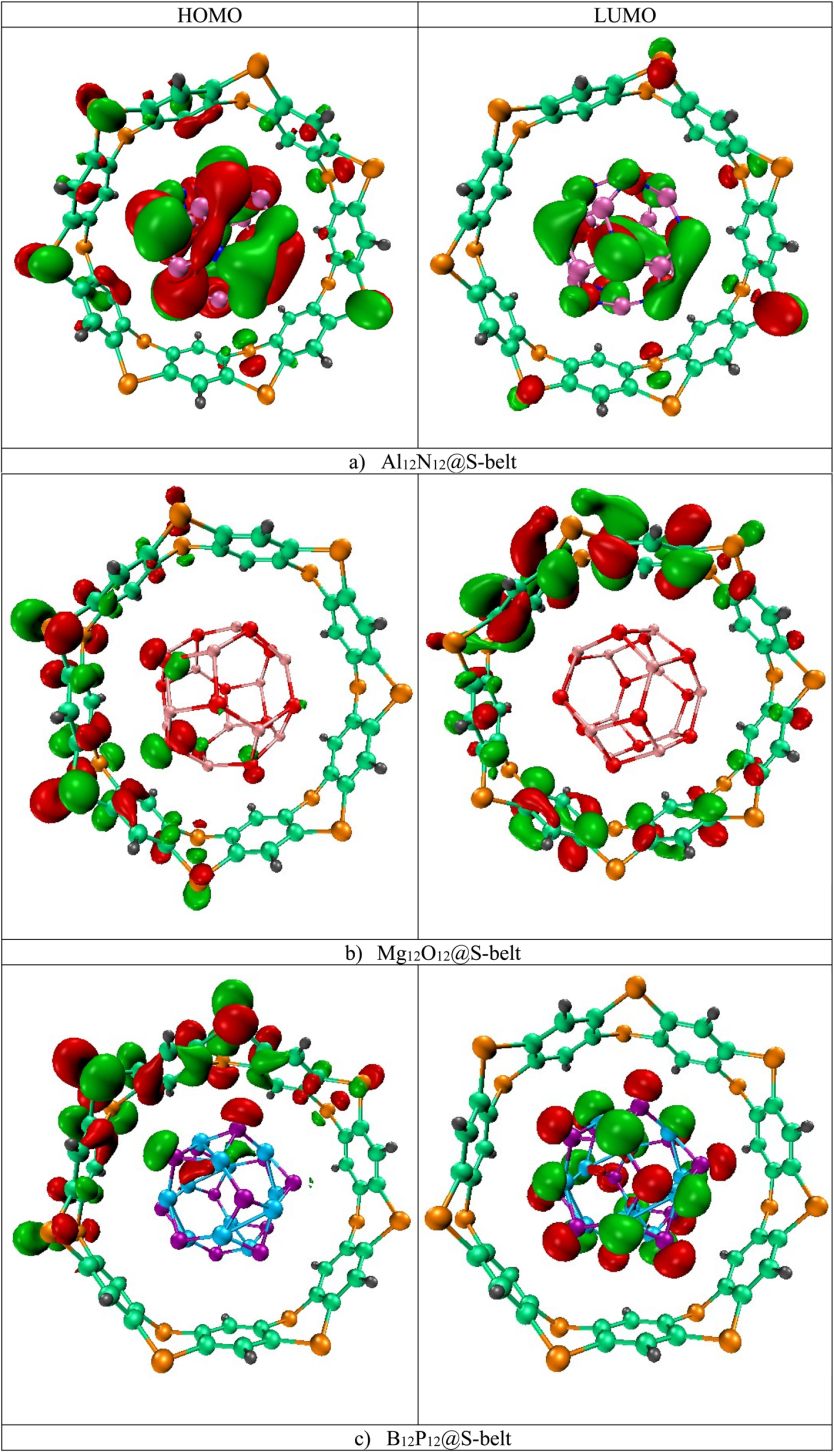
HOMO and LUMO isosurfaces for the (a) Al_12_N_12_@S-belt, (b) Mg_12_O_12_@S-belt, and (c) B_12_P_12_@S-belt complexes.

The HOMO, LUMO isosurfaces for the complexes are showcased in Fig. 2. Here, in the Al_12_N_12_@S-belt complex, the HOMO isosurfaces are present both on S-belt and Al_12_N_12_. This can be attributed to the proximity of HOMO levels of the bare host and guest species, resulting in the HOMO isosurfaces on both the species in the complex. As expected, the LUMO isosurfaces are present on Al_12_N_12_ in the complex. Moreover, the HOMO isosurfaces for the Mg_12_O_12_@S-belt complex mostly reside on the S-belt. The LUMO isosurfaces for this complex also reside on the S-belt. This might be due to the closeness in the LUMO levels of bare S-belt and Mg_12_O_12_, causing the LUMO isosurfaces to appear either on the S-belt or Mg_12_O_12_. The HOMO and LUMO isosurfaces for the B_12_P_12_@S-belt complex reside mostly on the S-belt and B_12_P_12_, respectively. Overall, the values of *E*_HOMO_, *E*_LUMO_ and *E*_gap_ of the complexes shows the fine tuning of the HOMO, LUMO levels and the energy gaps for all the three designed complexes, which is the main theme of the study.

### 3.3. PDOS analysis

**Fig. 3:**
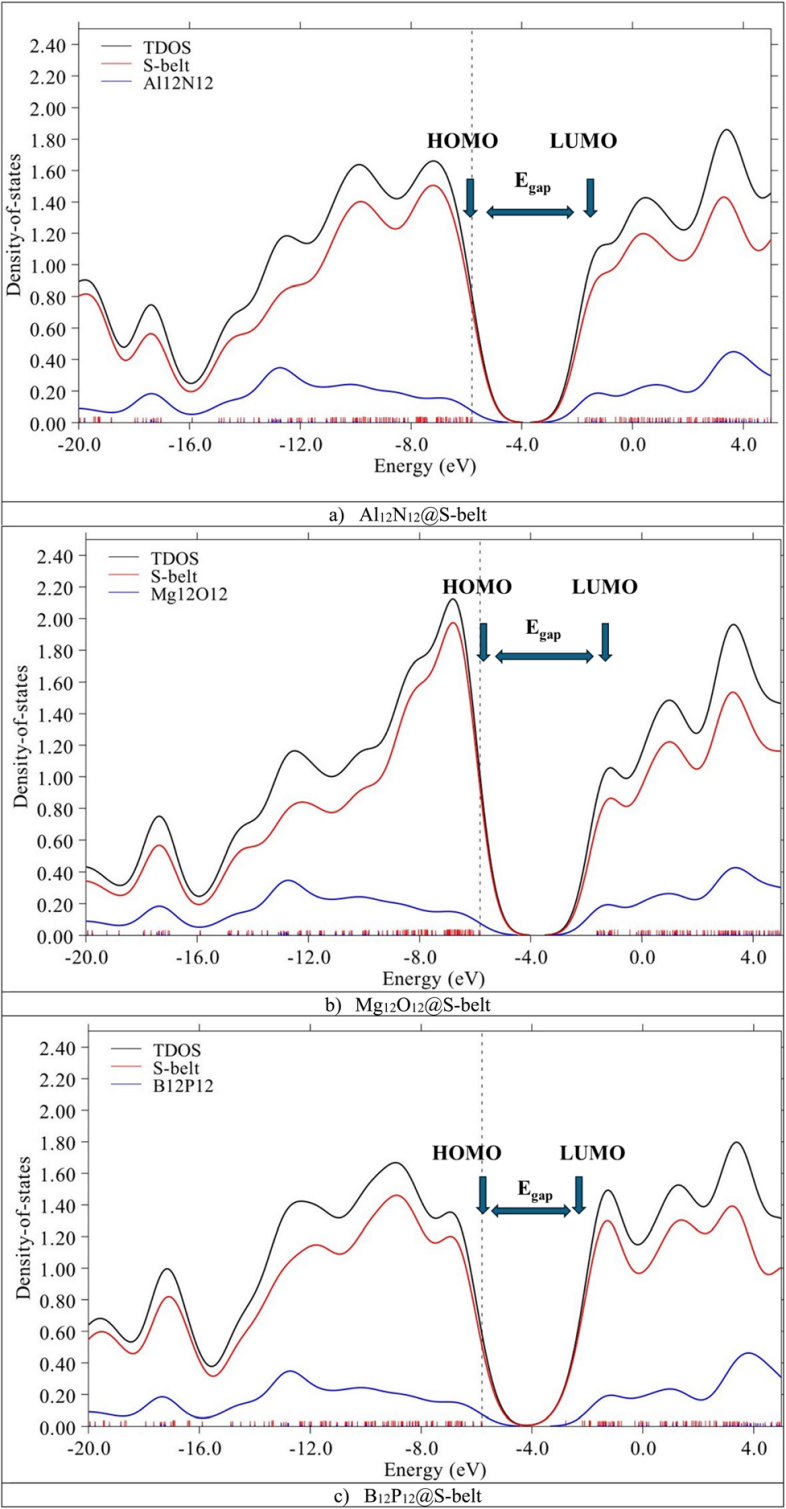
DOS spectra for the (a) Al_12_N_12_@S-belt, (b) Mg_12_O_12_@S-belt, and (c) B_12_P_12_@S-belt complexes.

The HOMO, LUMO levels and energy gaps obtained through FMO analysis are validated *via* PDOS analysis. Fig. 3 shows the DOS spectra for the complexes, with black, red, and blue colored curves showing the total density of states (TDOS) for the complexes, PDOS for host (S-belt) and guests (Al_12_N_12_, Mg_12_O_12_, and B_12_P_12_), respectively. In the DOS spectra of the Al_12_N_12_@S-belt complex, the energy of the HOMO of the complex is −5.80 eV, represented by a vertical dotted line. Similarly, the energy of the LUMO for the complex is −1.96 eV, represented by an arrow pointing towards the *x*-axis, after the HOMO. Similarly, the energy gap of the complex is 3.83 eV. Similar results were obtained for the other two complexes. The positions of HOMO, LUMO and *E*_gap_ are labelled for all the complexes. The contribution of HOMO and LUMO of the bare host and guest towards the HOMO and LUMO of the complexes, respectively, determined *via* FMO analysis is justified by the DOS spectra for all the designed complexes.

### 3.4. NBO analysis

Natural bond orbital (NBO) analysis was performed to investigate the direction of electron density shift after complex formation. It is observed that the direction of charge transfer for the two complexes is from the host towards the guest, whereas for the third complex, the direction of overall charge transfer is from the guest towards the host. The NBO charges on Al and N atoms of bare Al_12_N_12_ are 0.737|*e*| and −0.737|*e*|, respectively, making it an overall neutral species. Similarly, these charges on Mg are 0.901|*e*| (on four atoms) and 0.902|*e*| (on eight atoms) whereas there are −0.901|*e*| (on four atoms) and −0.902|*e*| (on eight atoms) on O in bare Mg_12_O_12_, thus making it neutral. The charges on B and P atoms are −0.119|*e*| and 0.119|*e*| before complexation, making B_12_P_12_ overall neutral. After complexation, the sum of charges for Al_12_N_12_ and B_12_P_12_ are −0.307|*e*| and −0.163|*e*|, respectively, showing that the electron density slightly shifts towards the guest species in these complexes. On the other hand, the sum of charges for the Mg_12_O_12_@S-belt complex is 0.064|*e*|, indicating a little amount of overall shift in the electron density from the guest towards the host. Compared to the former two complexes, the charge transfer behavior for the Mg_12_O_12_@S-belt complex seems to be different. This behavior (electron density shift towards the S-belt in the Mg_12_O_12_@S-belt complex) can be attributed to its unique behavior in FMO analysis as both the HOMO and LUMO isodensities reside over the belt. The maximum NBO charge is found for the guest species in the Al_12_N_12_@S-belt complex, corroborating with the highest interaction energy for the same complex. Overall, NBO analysis indicates that for the Al_12_N_12_@S-belt and B_12_P_12_@S-belt complexes, the direction of charge transfer is from the host towards the guest, whereas for the Mg_12_O_12_@S-belt complex, the charge transfer is from the guest towards the host.

### 3.5. EDD analysis

**Fig. 4:**
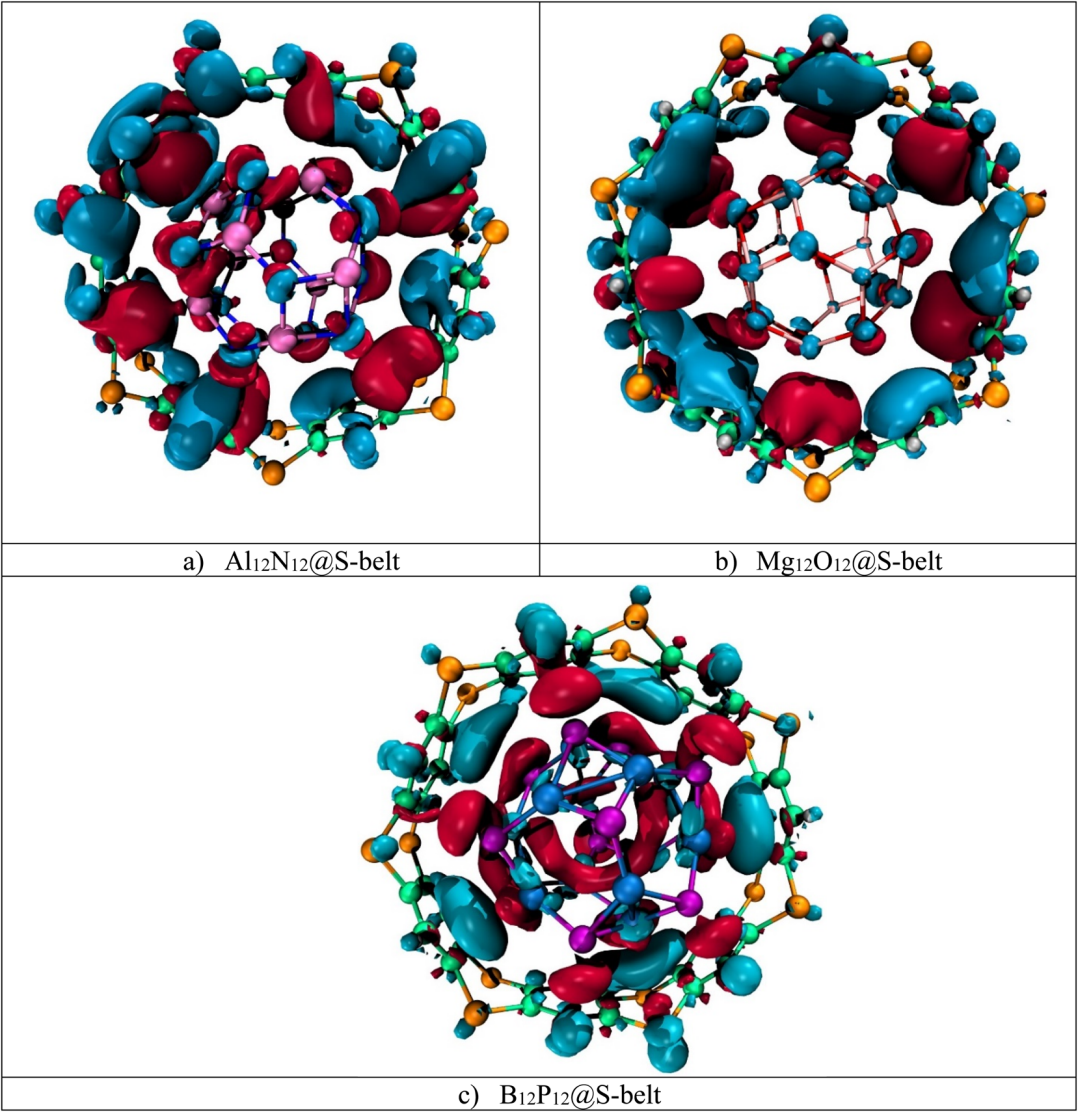
EDD isosurfaces for the (a) Al_12_N_12_@S-belt, (b) Mg_12_O_12_@S-belt, and (c) B_12_P_12_@S-belt complexes.

Electron density difference analysis (EDD) is the visual representation for the elaboration of NBO charge transfer. Fig. 4 shows the red and blue isosurfaces, demonstrating the regions of electron density depletion and accumulation, respectively. For the Al_12_N_12_@S-belt complex, the red and blue colored isosurfaces reside on both the host and guest species, illustrating the electron density transfer from the host towards the guest and *vice versa* (*i.e.*, electron donation and back-donation effect). Similarly, the other two complexes (*i.e.*, Mg_12_O_12_@S-belt and B_12_P_12_@S-belt) also show the electron donating and back-donating characteristics of the host and guest species. Overall, the greater red patches over the S-belt are observed for the Al_12_N_12_@S-belt and B_12_P_12_@S-belt complexes compared to the S-belt in the Mg_12_O_12_@S-belt complex. Moreover, a greater number of blue colored patches are present over the S-belt in case of the Mg_12_O_12_@S-belt complex. It shows that the charge is depleted from the host (S-belt) and is shifted towards the guests (*i.e.*, Al_12_N_12_ and B_12_P_12_) in the former two cases compared to the latter one. These findings validate the direction of electron density shift predicted through NBO charge analysis, *i.e.*, from the host to the guest for Al_12_N_12_ and B_12_P_12_ and guest to the host for the Mg_12_O_12_@S-belt complex.

### 3.6. NCI analysis

**Fig. 5:**
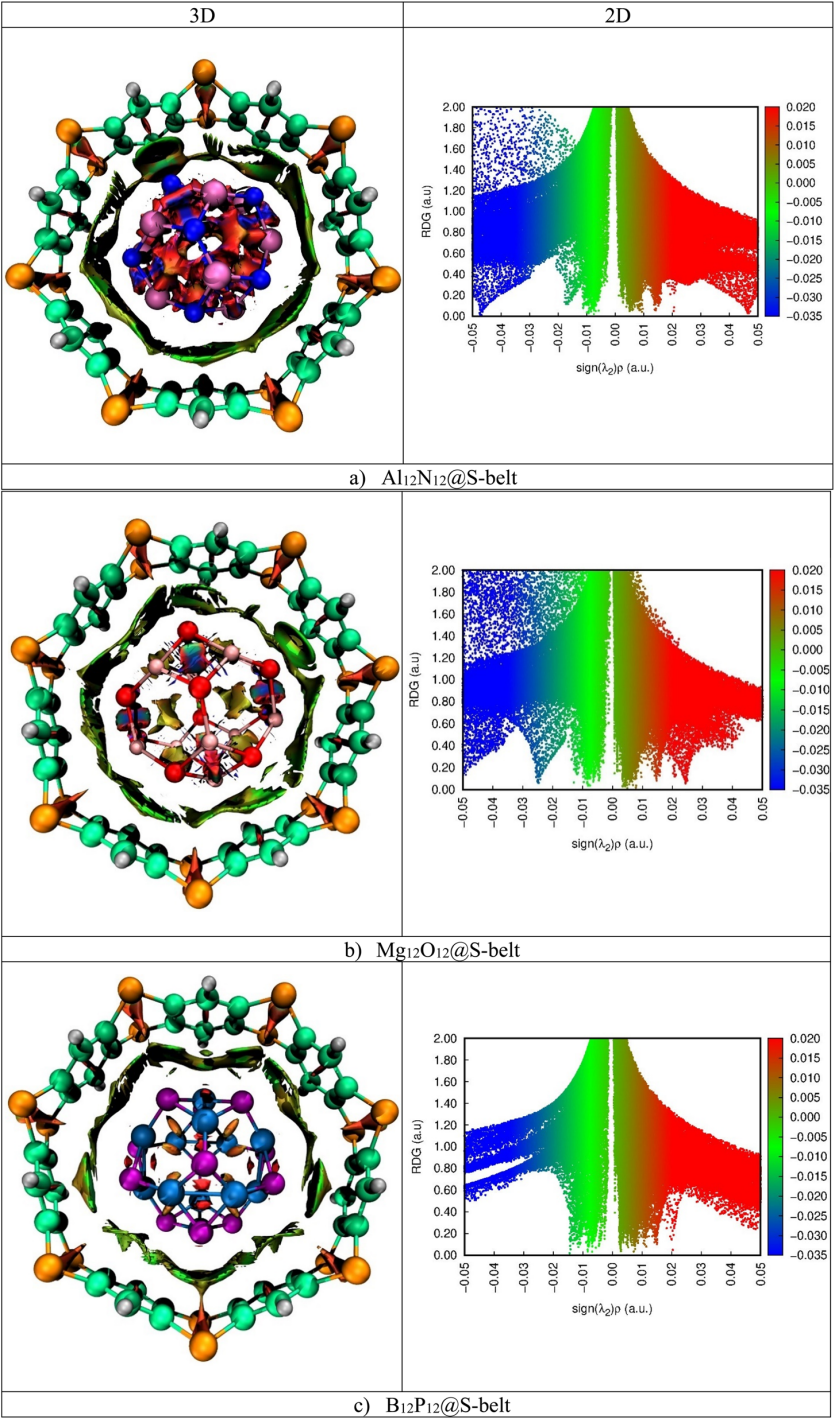
3D maps and 2D RDG plots for the (a) Al_12_N_12_@S-belt, (b) Mg_12_O_12_@S-belt, and (c) B_12_P_12_@S-belt complexes.

Noncovalent interaction index (NCI) analysis provides valuable information about the nature of interactions present in the complexes. The three-dimensional RDG maps and two-dimensional plots for the complexes are illustrated in Fig. 5. 3D maps show the colored isosurfaces demonstrating the nature of interactions, whereas 2D plots represent the colored spikes to delve into the nature and extent of interactions present between the host and guest species in the designed complexes. 2D RDG plots are generated based on variables of electron density (*ρ*) and reduced density gradient (∇^2^*ρ*).2
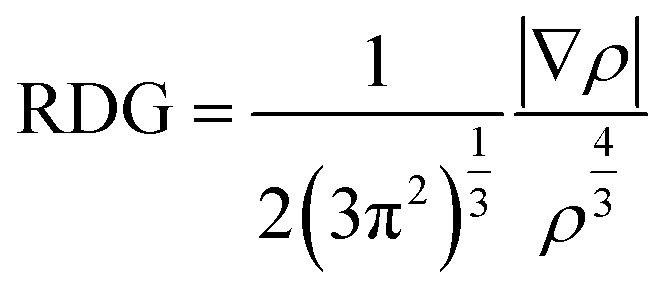
In the 2D plots of NCI, the values of sign(*λ*_2_)*ρ* range from −0.05 to 0.05 a.u. on the *x*-axis and on the *y*-axis, and RDG ranges from 0.00 to 2.00 a.u. The red spikes with sign(*λ*_2_)*ρ* values ranging from 0.02 to 0.05 a.u. represent the repulsive interactions, the blue spikes having sign(*λ*_2_)*ρ* in the range −0.02 to −0.05 a.u. indicate the electrostatic interactions, whereas the green spikes with values of sign(*λ*_2_)*ρ* ranging from −0.01 to 0.01 a.u. are an indication of the existence of van der Waals interaction.

Here, in the 3D map of the Al_12_N_12_@S-belt complex, it is evident that the green patches exist predominantly between the host and guest species, demonstrating that the van der Waals interactions are the reason behind the stability of the host–guest complexes. The red patches are present in 1,4-benzothiazine units of the S-belt as well as inside the Al_12_N_12_ cage, showing the presence of repulsive interactions there. Moreover, the blue patches are present inside the Al_12_N_12_ cage, specifying the presence of electrostatic forces of attraction. Similarly, in the 2D plot of the complex, the red, blue and green spikes are prominent, revealing the presence of the repulsive, electrostatic and van der Waals interactions, respectively. In the 3D map of the Mg_12_O_12_@S-belt complex, the green colored patches are present between the host and guest species as well as inside the guest, indicating the van der Waals interaction between the host and the guest. The red colored patches are present in the units of the S-belt (*i.e.*, 1,4-benzothiazine) and inside the Mg_12_O_12_ cage, showing the forces of repulsion operating there. The blue colored patches exist inside the Mg_12_O_12_ cage, illustrating the presence of electrostatic attraction inside the cage. Furthermore, in the B_12_P_12_@S-belt complex, the host and guest are stabilized through the van der Waals interactions, as revealed by the green colored patches existing between the host and guest species. The red patches are present in the individual 1,4-benzothiazine units of the S-belt and inside the B_12_P_12_ cage. In the 2D plot of the complex, the red and green spikes are notable compared to the blue spikes due to the presence of repulsive (inside the cage and 1,4-benzothiazine units of the belt) and van der Waals interactions (between the host and guest) and the absence of electrostatic interactions, respectively. The greater spikes and patches are observed for the Al_12_N_12_@S-belt and Mg_12_O_12_@S-belt complexes compared to the B_12_P_12_@S-belt complex, justifying the comparatively greater value of *E*_int_ for the former two complex. Overall, it is noted that all the designed nano-Saturn complex systems are stabilized *via* van der Waals interactions.

### 3.7. QTAIM analysis

**Fig. 6:**
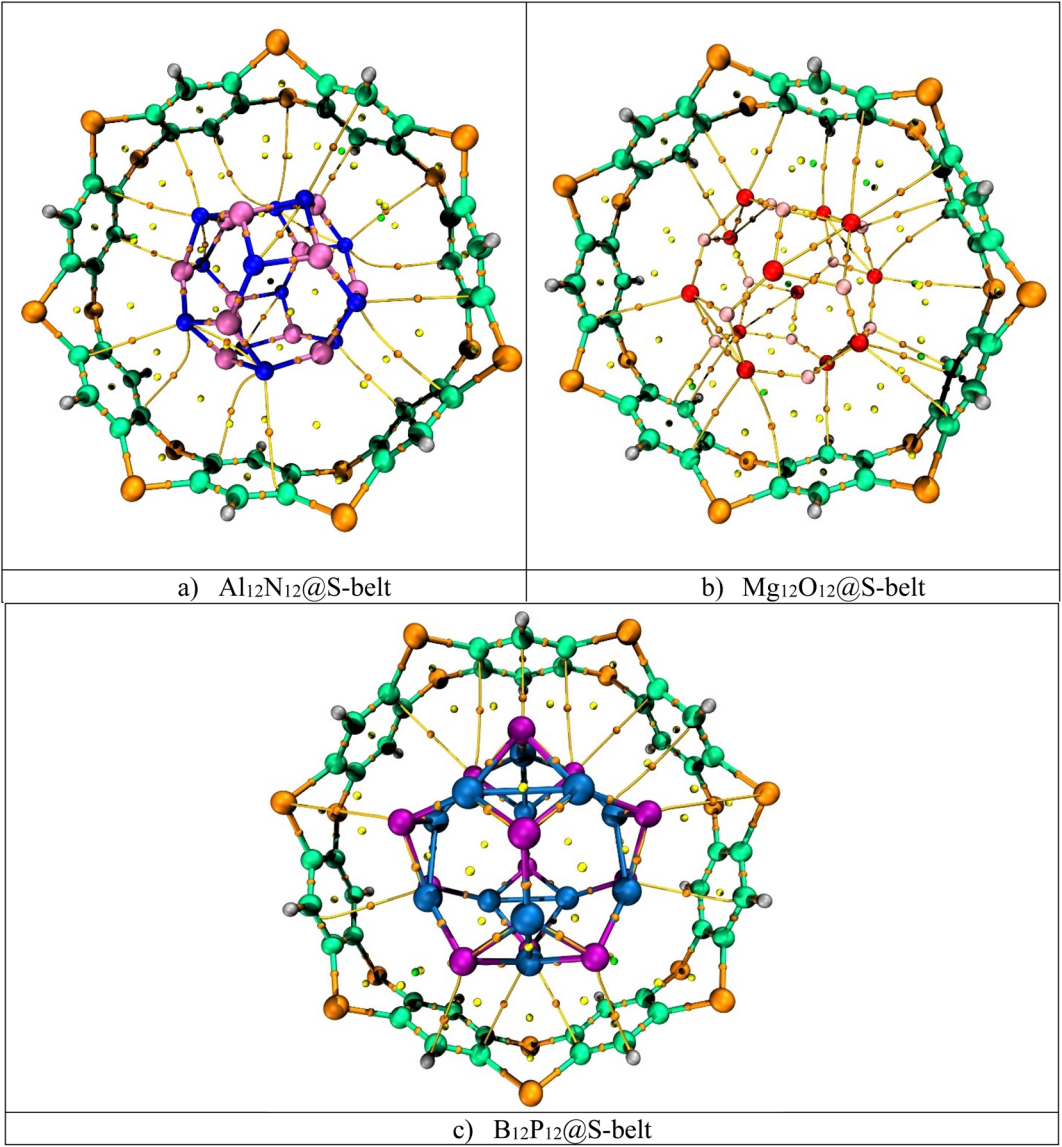
BCPs obtained through QTAIM for the (a) Al_12_N_12_@S-belt, (b) Mg_12_O_12_@S-belt, and (c) B_12_P_12_@S-belt complexes.

Quantum theory of atoms in molecule (QTAIM) is a topological analysis of electron density distribution. In order to understand the nature of interactions, the electron density *ρ*(*r*) and Laplacian of electron density ∇^2^*ρ*(*r*) are calculated along with other important parameters such as total energy density *H*(*r*), local kinetic energy *G*(*r*), and local potential energy *V*(*r*). The QTAIM topological parameters for the complexes are listed in Table 3, and the bond critical points (BCPs) are shown in Fig. 6. The Al_12_N_12_@S-belt complex shows 16 BCPs, whereas the Mg_12_O_12_@S-belt and B_12_P_12_@S-belt complexes show 18 and 14 BCPs, respectively. Table 3 shows that the values of *ρ*(*r*) are greater than 0.1, implying the presence of nonbonding (van der Waals) interactions. The values of ∇^2^*ρ*(*r*) and *H*(*r*) for all the complexes are positive, justifying the presence of van der Waals interactions. The values of −*V*/*G* for the Al_12_N_12_@S-belt, Mg_12_O_12_@S-belt and B_12_P_12_@S-belt complexes are in the range of 0.12–1.04, 0.81–2.29, and 0.70–0.89 a.u., respectively. The lower values of −*V*/*G* verify the presence of nonbonding interactions. Furthermore, the interaction energy values for individual bonds are less than 3, *i.e.*, signifying the presence of van der Waals interactions. The BCPs and *E*_int_ of individual bonds for the Al_12_N_12_@S-belt and Mg_12_O_12_@S-belt complexes are greater compared to the B_12_P_12_@S-belt complex. Overall, these results are in corroboration with the NCI analysis, demonstrating that the main reason for the stability of the nano-Saturn host–guest complexes is the nonbonding or van der Waals interactions.

**Table 3:** QTAIM parameters for the Al_12_N_12_@S-belt, Mg_12_O_12_@S-belt, and B_12_P_12_@S-belt complexes

Complexes	Ana-belt	CPs	*ρ* (a.u)	∇^2^*ρ* (a.u)	*G*(*r*) (a.u)	*V*(*r*) (a.u)	*H*(*r*) (a.u)	−*V*/*G*	*E*_int_ (kcal mol^−1^)
Al_12_N_12_@S-belt	N8–C36	132	0.0086	0.025	0.0055	−0.0049	0.0007	0.89	1.54
	N6–C58	157	0.0113	0.026	0.0061	−0.0058	0.0004	0.95	1.82
	N2–C50	193	0.0085	0.025	0.0054	−0.0046	0.0008	0.85	1.44
	N2–C46	214	0.0093	0.026	0.0057	−0.0050	0.0007	0.88	1.57
	N1–C45	223	0.0081	0.016	0.0039	−0.0037	0.0002	0.95	1.16
	N1–C44	244	0.0058	0.017	0.0036	−0.0030	0.0007	0.83	0.94
	Al14–C31	249	0.0175	0.038	0.0112	−0.0013	−0.0017	0.12	0.41
	N3–C28	262	0.0099	0.028	0.0064	−0.0056	0.0008	0.88	1.76
	N11–C32	247	0.0069	0.020	0.0044	−0.0037	0.0007	0.84	1.16
	N11–C76	229	0.0101	0.017	0.0045	−0.0047	−0.0002	1.04	1.47
	N4–C75	224	0.0085	0.025	0.0055	−0.0048	0.0007	0.87	1.51
	N4–C69	197	0.0065	0.018	0.0039	−0.0033	0.0006	0.85	1.04
	N12–C71	167	0.0113	0.033	0.0073	−0.0064	0.0009	0.88	2.01
	N9–C64	134	0.0080	0.023	0.0051	−0.0044	0.0006	0.86	1.38
	N9–C63	123	0.0075	0.014	0.0035	−0.0033	0.0001	0.94	1.04
	N8–C38	121	0.0075	0.016	0.0037	−0.0034	0.0003	0.92	1.07
Mg_12_O_12_@S-belt	O24–C77	256	0.0100	0.032	0.0071	−0.0063	0.0008	0.89	1.98
	Mg3–C76	261	0.0079	0.033	0.0069	−0.0056	0.0013	0.81	1.76
	O23–C76	249	0.0082	0.019	0.0045	−0.0043	0.0002	0.96	1.35
	O23–C73	223	0.0076	0.022	0.0049	−0.0041	0.0007	0.84	1.29
	Mg1–C71	197	0.0084	0.036	0.0075	−0.0061	0.0014	0.81	1.91
	O21–C71	186	0.0079	0.018	0.0044	−0.0044	0.0002	1.00	1.38
	O21–C65	174	0.0087	0.029	0.0064	−0.0054	0.0009	0.84	1.69
	O20–C63	142	0.0130	0.038	0.0090	−0.0084	0.0005	0.93	2.64
	O19–C58	124	0.0064	0.016	0.0035	−0.0031	0.0004	0.88	0.97
	O19–C39	119	0.0090	0.029	0.0064	−0.0055	0.0008	0.86	1.72
	O18–C37	132	0.0097	0.024	0.0024	−0.0055	0.0003	2.29	1.72
	Mg10–C37	131	0.0089	0.037	0.0037	−0.0065	0.0013	1.76	2.04
	O17–C55	144	0.0079	0.026	0.0026	−0.0049	0.0008	1.88	1.54
	O16–C45	223	0.0076	0.022	0.0022	−0.0041	0.0007	1.86	1.29
	O14–C44	239	0.0125	0.040	0.0040	−0.0085	0.0008	2.12	2.67
	O14–C27	253	0.0085	0.026	0.0026	−0.0050	0.0008	1.92	1.57
	O13–C31	271	0.0092	0.030	0.0030	−0.0054	0.0011	1.80	1.69
	O24–C29	263	0.0064	0.020	0.0020	−0.0035	0.0008	1.75	1.10
B_12_P_12_@S-belt	P18–C78	246	0.0116	0.033	0.0072	−0.0061	0.0011	0.85	1.91
	B1–C79	230	0.0073	0.020	0.0042	−0.0033	0.0009	0.78	1.04
	P16–S72	212	0.0057	0.015	0.0031	−0.0023	0.0008	0.74	0.72
	P18–C68	178	0.0145	0.043	0.0095	−0.0083	0.0012	0.87	2.60
	P14–C60	122	0.0069	0.022	0.0043	−0.0030	0.0013	0.70	0.94
	P20–C58	123	0.0131	0.037	0.0082	−0.0073	0.0010	0.89	2.29
	P20–C39	118	0.0125	0.035	0.0078	−0.0069	0.0010	0.88	2.16
	P22–H84	111	0.0074	0.024	0.0046	−0.0033	0.0013	0.72	1.04
	P24–C53	153	0.0142	0.043	0.0094	−0.0080	0.0014	0.85	2.51
	P28–S52	190	0.0061	0.016	0.0033	−0.0025	0.0008	0.76	0.78
	P24–C43	235	0.0118	0.034	0.0073	−0.0062	0.0011	0.85	1.94
	P24–C26	247	0.0079	0.023	0.0048	−0.0038	0.0010	0.79	1.19
	B8–C28	242	0.0090	0.024	0.0052	−0.0045	0.0007	0.86	1.41
	P18–C29	252	0.0084	0.024	0.0051	−0.0041	0.0010	0.80	1.29

### 3.8. Absorption analysis

**Fig. 7:**
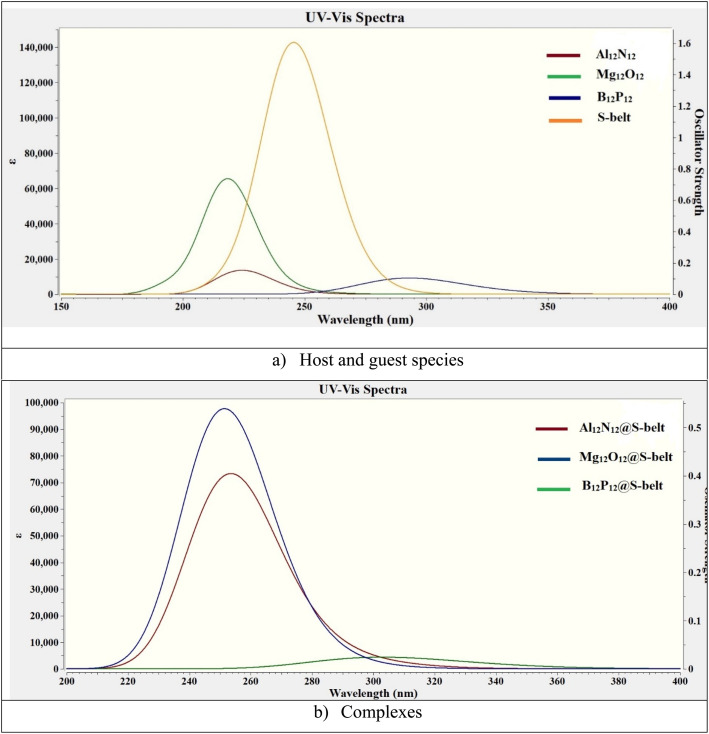
UV-vis absorption spectra for the bare (a) host and guest species, and (b) complexes.

The UV-vis absorption analysis of the individual host, guest species and the complexes is performed and the absorption maxima (*λ*_max_), excitation energies (Δ*E*), and oscillator strengths (*f*_o_) are reported in Table 4. The absorption spectra of the individual components and the complexes are showcased in Fig. 7. The excitation energy values (Δ*E*) for the S-belt and the nanocages are greater, ranging from 4.24 to 5.66 eV. This results in maximum absorption at smaller wavelength, *i.e.*, in the UV region (219–292 nm). The oscillator strength of the S-belt (host) is higher than that of the nanocages (guests). After the host–guest complex formation, a minor decrease in the values of excitation energies is seen (4.08–4.93 eV). Due to this decrease, the absorption maxima of the complexes is slightly increased (251–304 nm). The bathochromic or red shift is observed for all the complexes, but the region of maximum absorption still remains the same, *i.e.*, the UV region. The oscillator strength of the B_12_P_12_@S-belt complex is minimum compared to the other two complexes due to the minimum oscillator strength of the bare B_12_P_12_. Hence, it can be concluded from absorption analysis that all the complexes show maximum absorption in the UV region.

**Table 4:** Absorption analysis parameters, *i.e.*, excitation energies (Δ*E*), absorption maxima (*λ*_max_) and oscillator strength (*f*_o_) for the Al_12_N_12_@S-belt, Mg_12_O_12_@S-belt, and B_12_P_12_@S-belt complexes

Compounds	Δ*E* (eV)	*λ*_max_ (nm)	*f*_o_ (a.u)
S-belt	5.04	246	1.62
Al_12_N_12_	5.56	223	0.10
Al_12_N_12_@S-belt	4.89	254	0.52
Mg_12_O_12_	5.66	219	0.44
Mg_12_O_12_@S-belt	4.93	251	0.99
B_12_P_12_	4.24	292	0.06
B_12_P_12_@S-belt	4.08	304	0.03

## 4. Conclusion

In summary, three supramolecular complexes are designed (Al_12_N_12_@S-belt, Mg_12_O_12_@S-belt, and B_12_P_12_@S-belt) by encapsulating the fullerene-like nanostructures inside the S-belt. The greater values of *E*_int_ up to 63.64 kcal mol^−1^ indicate the stability of the complexes. FMO analysis demonstrates the greater role of HOMO of the host (S-belt) and LUMO of the guests (Al_12_N_12_, Mg_12_O_12_, and B_12_P_12_) towards the HOMO and LUMO levels of the designed supramolecular complexes, respectively. The energies of the HOMO and LUMO of the complexes resemble the energy of HOMO of the host and energy of LUMO of the guests, respectively. Compared to those of bare host and guest species (3.74–514 eV), the energy gap of the complexes also experience a slight decrease, *i.e.*, 3.02–4.20 eV. PDOS spectra analysis further clarifies the FMO. Furthermore, the negative NBO charges on the guest species of the two complexes show the electron density shift towards the guest species, whereas for one of the complexes, the magnitude of NBO charge on the guest species is positive, indicating the electron density shift towards the host (S-belt). The highest NBO charge of −0.307|*e*| is observed for the guest species encapsulated inside the S-belt in the Al_12_N_12_@S-belt complex, corroborating with the highest *E*_int_ for the complex. EDD verifies the NBO analysis. NCI and QTAIM analyses explain that the host–guest supramolecular complexes are stabilized *via* van der Waals interactions. The results of absorption analysis shows that the bare host and guests as well as the designed complexes show absorption maxima in the UV region range of 251–304 nm. Overall, this work highlights the design of stable complexes and controlled tuning of the HOMO and LUMO levels over the distinct species in the complexes. This study opens the doors for the controlled tuning of electronic properties and the design of other similar systems with aligned HOMO, LUMO levels for applications in optoelectronic devices.

## Data availability

Data will be made available on request.

## Conflicts of interest

There are no conflicts to declare.

## Supplementary Material

RA-014-D4RA07068B-s001
