# Supplementary material for: Controlled tuning of HOMO and LUMO levels in supramolecular nano-Saturn complexes
Source: RSC Adv. 2024 Dec 12;14(53):39395–407. doi: 10.1039/d4ra07068b (PMC11636639; doi:10.1039/d4ra07068b)
Supplement: RA-014-D4RA07068B-s001 [file RA-014-D4RA07068B-s001.pdf]

Supplementary Information (SI) for RSC Advances.
This journal is © The Royal Society of Chemistry 2024

Supporting information

Controlled tuning of HOMO and LUMO levels in supramolecular nano-Saturn complexes

Maria Maqbool[a] & *Khurshid Ayub*[a]*

[a] Department of Chemistry, COMSATS University, Abbottabad Campus, KPK, Pakistan 22060

*Corresponding author.

Tel: +92-992-383591.

E-mail: khurshid@cuiatd.edu.pk (K.A.)

Density Functional Theory

The density functional theory is quantum mechanical method which is used in chemistry to study the electronic structure of many body systems. The theory was developed in 1960. DFT defines many body problems by considering no interaction between the electrons [1]. ωB97XD is a range-separated version of Becke's 97 functional, with 22% Hartree-Fock exchange at short range and 100% Hartree-Fock at long range. ωB97XD is particularly useful for systems where dispersion interactions are critical, such as in studying molecular complexes, protein-ligand interactions, and other weakly bound systems [2]. The Hohenberg-Kohn theorem was published in 1964 which set the foundation of the time dependent density functional theory TD-DFT. According to this theorem, the density ρ (r) of the particle determines the external potential V (r) of the particle. The many particles ground state is the functional of density ρ (r) of the particle. The correct estimation of the density of the ground state of the particle minimizes the overall energy of the system.

This theory explains that the time dependent density of the system is enough to understand the time dependency of the functional rather than exchange correlation. This theory is applicable for open shell systems as well as restricted and unrestricted spin properties. Moreover, the TD-DFT calculations on our systems are performed by taking 30 excited states into consideration.

Valence electrons play a crucial role in most chemical reactions. Therefore, it is necessary to present these valence orbitals through multiple basis functions. In split valence basis sets, core and valence electrons are treated independently by separated Gaussian functions, due to which these basis sets are known as split valence basis sets. Core electrons do not require flexibility because core electrons are not much affected by chemical reactions, therefore, core electrons are presented with single zeta. Whereas valence electrons are mainly involved in the chemical reactions, therefore, valence orbitals are described through double, triple, or quadruple zeta basis sets. The 6-31G has two parts, inner shell electrons are represented through one contracted Gaussian function "6", which consists of six primitive Gaussian functions. Similarly, the outer shell is presented with two contracted Gaussian function "31", where "3" and "1" stand for valence contracted Gaussian which are called three and one primitive Gaussian functions, respectively. The polarization functions enhance the flexibility of the valence electrons. The polarization function "p" explains the dispersion of "s" orbitals, whereas d and f basis function

containing polarization functions describe the flexibility of “p” and “d” valance orbitals, respectively [3].

The RDG, or real-space density gradient, is a crucial metric to evaluate the spatial distribution of electronic density within molecules. RDG or NCI analysis provides valuable information about the spatial distribution of electrons and the strength of the interactions present between atoms. The NCI analysis provides an index, based on electron density and derivatives of electronic density, that enables identification of noncovalent interactions. It is based on a 2D plot of the reduced density gradient, s, and the electron density, ρ, where:

$$RDG = \frac{1}{2(3\pi^2)^{\frac{1}{3}}} \frac{|\nabla\rho|}{\rho^{\frac{4}{3}}} \quad (1)$$

Based on the divergence theorem, the sign of the Laplacian ($\nabla^2\rho$) of the density determines whether the net gradient flux of density is entering ($\nabla^2\rho < 0$) or leaving ($\nabla^2\rho > 0$) an indefinitely small volume around a reference point. The sign of $\nabla^2\rho$ tells whether the density is concentrated or depleted at that point, compared to surroundings. The sign of the Laplacian alone cannot reliably distinguish different types of weak interactions because it is dominated by negative contributions from nuclei. Instead, analyzing the eigenvalues (λ_i) of the electron-density Hessian, which describe variations along principal axes, provides greater insight. These eigenvalues are related to the Laplacian through the equation $\nabla^2\rho = \lambda_1 + \lambda_2 + \lambda_3$, where $\lambda_1 < \lambda_2 < \lambda_3$. At the nuclei, all eigenvalues are negative, but moving away from nuclei, λ_3 becomes positive. In molecules, λ_3 varies along the internuclear axis, while λ_1 and λ_2 reflect density changes in the plane perpendicular to this axis. The second eigenvalue, λ_2, is particularly important for the identification of the type of interactions. Bonding interactions, such as hydrogen bonds, show density accumulation perpendicular to the bond, resulting in $\lambda_2 < 0$. In contrast, non-bonded interactions, such as steric repulsion, lead to density depletion, giving $\lambda_2 > 0$. van der Waals interactions, which involve negligible density overlap, typically result in $\lambda_2 \lesssim 0$. Therefore, the sign of λ_2 can distinguish between interaction types, while the overall electron density provides a measure of interaction strength.

Moreover, Bader's quantum theory of atoms in molecules (QTAIM) analysis is carried out to examine the nature of interactions among the host-guest complexes. In QTAIM, the total electron density ρ(r), Laplacian of electron density $\nabla^2\rho(r)$, total electron energy density H(r), local kinetic energy G(r), and local potential energy V(r) are some of the key parameters, signifying the strength and nature of interaction at bond critical points (BCPs). Here, the total electron energy density H(r), is obtained by adding the local kinetic and potential energies *i.e.,* V(r) and G(r), respectively.

$$H(r) = V(r) + G(r) \tag{2}$$

The values of the QTAIM parameters clearly reveal the kind of interactions between the components of host-guest complexes. The positive values of H(r) and $\nabla^2\rho(r)$ demonstrate the non-covalent interactions between the host and guest molecules, whereas the negative values prove the presence of covalent bonding. Furthermore, H(r) > 0 illustrates closed-shell interactions, while H(r) < 0 explains the shared shell interactions. The strength of non-covalent interactions is determined by ρ(r). The values of ρ(r) < 0.1, are the result of the weak non-covalent interactions, whereas the positive values ρ(r) > 0.1, result from the strong covalent interactions. The values of -V/G < 1, show the existence of non-covalent interactions between the host-guest complexes. The nature of bonding can be better understood by evaluating the interaction energy of individual bonds, *i.e.,*

$$E_{int} = \frac{1}{2}V(r) \tag{3}$$

When the values of E_{int} lie in the range of 3-10 kcal/mol, strong electrostatic interactions are expected in the host-guest complexes. On the other hand, the values of the E_{int} less than 3 kcal/mol are indicative of the existence of weak van der Waals forces.

Cartesian coordinates of optimized complexes

1. $Al_{12}N_{12}$@S-belt

N	1.68901700	2.09761800	2.10389700
N	2.77636400	0.27881600	-0.35198300
N	0.51217400	2.50087900	-0.86956700
N	-2.40514000	1.16857100	-0.61053700
N	-0.13910100	-0.08124600	3.66607500
N	2.45171000	-1.04426000	2.00259400
N	0.18933400	0.20629600	-2.30166600
N	1.04245100	-2.39648300	-0.57896400
N	-1.63350500	-1.94626500	-0.71341200
N	-0.47321600	-2.40648600	2.27210700
N	-0.99297000	2.54475100	1.97393800
N	-2.73312300	-0.15295400	1.74401400
Al	1.53815000	1.05763900	-1.36000000
Al	0.48778700	2.84097900	0.89217500
Al	-0.88274100	1.47155800	-1.47755800
Al	-2.24314100	1.54453900	1.19310100
Al	0.16966500	1.54261000	2.98653500
Al	2.58209900	0.71069600	1.43093500
Al	2.31633400	-1.41888700	0.19063500
Al	0.92963100	-1.35955200	2.86781900
Al	-0.43406700	-2.65993400	0.50015600
Al	-1.49304300	-0.93938500	2.74744400
Al	-0.11792800	-1.41001000	-1.60911900
Al	-2.56282700	-0.57942400	-0.05045500
S	3.52695600	5.16814800	-1.94595500
C	2.02674300	5.36160900	-1.02010000

C	2.03311300	5.32003200	0.37811700
C	0.82496900	5.51357500	-1.70303000
C	-0.36969300	5.69677700	-1.01336200
C	-0.38200900	5.65497600	0.38421200
C	0.81632200	5.40621000	1.07536300
S	-1.88144200	5.91851200	1.29385800
S	3.55337800	5.15590300	1.27756900
S	4.26197600	-4.58827300	1.27654600
C	2.80057600	-5.05548700	0.37914600
C	2.79039500	-5.07907200	-1.01818400
C	1.64344900	-5.37569600	1.08658700
C	0.50008600	-5.79624700	0.40729100
C	0.49693400	-5.82870900	-0.99328300
C	1.63068200	-5.43751400	-1.69780000
S	-0.95584700	-6.32675100	-1.88767100
S	4.22128400	-4.60853900	-1.96201400
C	4.28027700	3.83505400	-1.04339200
C	4.30532900	3.83748700	0.35361000
C	4.92409200	2.78909800	1.03523100
C	5.56527700	1.77172800	0.32828000
C	5.53254800	1.76602100	-1.06893100
C	4.86545600	2.78216100	-1.74665100
S	6.35520900	0.45326900	1.22440000
C	5.74891100	-0.95140700	0.31690900
C	5.72097800	-0.95185000	-1.08075800
S	6.29561400	0.45696500	-1.99733200
C	5.24057900	-2.03977900	1.02139900
C	4.77559300	-3.16774900	0.33944500

C	4.75795200	-3.17362300	-1.06302900
C	5.19679600	-2.04787300	-1.76359700
S	-1.86515700	5.94408600	-1.93451500
C	-2.18655200	-5.40986800	-0.99186600
C	-2.17415800	-5.37226500	0.40881400
C	-3.15626000	-4.70257700	-1.69430700
C	-4.15909600	-4.02061400	-1.01257100
C	-4.15628700	-3.98631000	0.38483200
C	-3.13804200	-4.62670300	1.08990100
S	-5.40433200	-3.17230100	-1.95292600
C	-5.51174900	-1.64475700	-1.05287800
C	-5.55136600	-1.63269800	0.34806200
S	-5.43063500	-3.13599100	1.28699500
C	-5.58601400	-0.44044200	-1.75780700
C	-5.77759800	0.76189500	-1.07925800
C	-5.82800000	0.76970800	0.31821800
C	-5.67581200	-0.41954500	1.02568000
S	-5.90509300	2.27544800	-1.99914400
S	-5.99491100	2.29611900	1.21692800
C	-4.81766300	3.29584900	0.33319300
C	-4.78783800	3.29150200	-1.06567800
C	-3.89153600	4.06275900	1.04442300
C	-2.98982900	4.88738600	0.36557100
C	-2.97474000	4.89005100	-1.03094400
C	-3.84926000	4.06579000	-1.73785500
S	-0.94630800	-6.27690100	1.32683200
H	0.81596900	5.46795000	-2.78631600
H	0.81274400	5.36255100	2.16151900

H	1.62483500	-5.28932100	2.16772100
H	1.60334900	-5.39098200	-2.78079500
H	4.90265000	2.77294300	2.12078700
H	5.11625500	-2.02266100	-2.84471200
H	-3.12147700	-4.66901400	-2.77744800
H	-5.48383800	-0.44173000	-2.83734100
H	-5.60519900	-0.39936800	2.10600700
H	-3.87847300	4.03744700	2.12999800
H	5.16579000	-2.00115500	2.10149700
H	-3.08792600	-4.54569200	2.17033100
H	-3.79130000	4.02137800	-2.82024600
H	4.79723000	2.75378100	-2.82934100

2. $Mg_{12}O_{12}$@S-belt

Mg	2.93009400	-0.39503700	-1.20109600
Mg	0.99008300	1.19211600	-3.29929400
Mg	1.58499300	2.52428200	-1.05895300
Mg	-0.50378300	2.05636700	1.38600900
Mg	1.74346500	0.65942200	1.62011700
Mg	-1.57856500	2.32842200	-1.69256500
Mg	-2.88990600	0.48672000	-0.25156500
Mg	-1.71849800	-0.56830200	-3.07386800
Mg	0.55238100	-1.93692400	-2.82534600
Mg	-1.53263600	-2.44759800	-0.40688000
Mg	-0.92339100	-1.08152700	1.81638000
Mg	1.64523800	-2.26558400	0.21986400
O	0.14781300	2.67844800	-2.37798600
O	-1.95024300	2.12118700	0.19898600

O	0.05613700	-0.35633400	-3.84340000
O	-2.67205200	0.78859300	-2.17353900
O	-2.46778500	-1.11282000	0.65362600
O	-1.27720000	-2.28461700	-2.28028800
O	-0.08543900	-2.56659100	0.89588200
O	2.04123800	-2.05792400	-1.68338600
O	2.66978500	-0.69567900	0.70787300
O	-0.02248200	0.47124900	2.41596300
O	2.53572300	1.20338800	-2.12952400
O	1.31533400	2.36031000	0.80607700
S	-3.36204500	5.21623200	1.93601700
C	-1.83056500	5.30399000	1.04087000
C	-1.80283700	5.28922400	-0.35684100
C	-0.63565400	5.41540500	1.75219000
C	0.57281100	5.60926900	1.07951200
C	0.58976600	5.62699900	-0.31606900
C	-0.58999300	5.41687300	-1.02954900
S	2.10554100	5.90779600	-1.20866400
S	-3.29083300	5.10631000	-1.32142300
S	-4.50934200	-4.41693100	-1.25170400
C	-3.03519000	-4.83460300	-0.34796900
C	-3.03061000	-4.85252500	1.05490100
C	-1.87146700	-5.16930900	-1.04899600
C	-0.73499800	-5.60213400	-0.36237900
C	-0.73821200	-5.62625800	1.03473200
C	-1.87087900	-5.21701700	1.73499600
S	0.69170400	-6.14173100	1.94890800
S	-4.48609200	-4.41822800	1.97620600

C	-4.22180800	3.95206100	1.03119400
C	-4.22124900	3.92588000	-0.36496200
C	-4.89733900	2.91629200	-1.04390600
C	-5.60562700	1.94635300	-0.33399400
C	-5.58181300	1.95382100	1.06710400
C	-4.87319700	2.94783900	1.74070100
S	-6.49916100	0.68587600	-1.21594200
C	-5.90922800	-0.73584500	-0.32240800
C	-5.89599700	-0.73987600	1.07829600
S	-6.42816000	0.69147000	1.99224700
C	-5.44093100	-1.84735600	-1.02205100
C	-5.04006800	-2.98973500	-0.33272600
C	-5.03373700	-2.99317900	1.06493100
C	-5.42789100	-1.85666800	1.76385100
S	2.06728300	5.83735200	2.01057100
C	1.98305900	-5.33796000	1.03552400
C	2.00077700	-5.34571500	-0.36539200
C	3.00507800	-4.69309100	1.73402700
C	4.08886900	-4.13724500	1.04813300
C	4.11318000	-4.16016100	-0.35054500
C	3.04764500	-4.72809400	-1.04970000
S	5.41266300	-3.38689900	1.96288000
C	5.58151500	-1.87596600	1.04545100
C	5.59212200	-1.87864000	-0.35199500
S	5.45043700	-3.40689900	-1.25027600
C	5.66127000	-0.67373600	1.73818800
C	5.81148100	0.52760600	1.05559000
C	5.82291600	0.54003000	-0.34352800

C	5.68442400	-0.66451200	-1.04377100
S	5.97682200	2.02853700	1.99093900
S	6.01719600	2.06475000	-1.23495300
C	4.90276400	3.10401500	-0.32027000
C	4.88638200	3.09198900	1.07684400
C	4.03464100	3.95377100	-1.00976600
C	3.19472200	4.82333400	-0.30841100
C	3.17424000	4.79674400	1.09112400
C	4.00313800	3.91499100	1.77340300
S	0.69089400	-6.10326200	-1.29765400
H	-0.64742500	5.37521800	2.83716600
H	-0.54811000	5.32867000	-2.10966000
H	-1.84967900	-5.06407200	-2.12769900
H	-1.84325000	-5.16612000	2.81775200
H	-4.83850500	2.84328300	-2.12431300
H	-5.35279700	-1.83365100	2.84523600
H	2.95732600	-4.62156100	2.81508700
H	5.58683200	-0.67284500	2.82012400
H	5.67666800	-0.65747700	-2.13146500
H	4.01225700	3.92754700	-2.09487300
H	-5.38776100	-1.81928600	-2.10504900
H	3.01765000	-4.64885600	-2.12864300
H	3.94719000	3.85243600	2.85481400
H	-4.81026100	2.92357300	2.82317500

3. B12P12@S-belt

B	2.36361400	1.03074200	0.61510600
B	2.41259200	-0.91743800	1.95747900

B	1.11472800	1.36748700	3.26296600
B	1.20399900	-1.33036800	-0.76547600
B	0.02377900	-1.35389400	3.63252200
B	0.08011300	-2.67380000	1.68358400
B	-0.02833100	1.23268200	-1.12362300
B	-0.08894400	2.61238600	0.83400900
B	-2.36533100	-1.07502700	1.92165700
B	-1.24082500	1.28862200	3.24567000
B	-1.09375600	-1.40617200	-0.78323400
B	-2.42657900	0.87197300	0.57858900
P	2.76127100	-0.73908400	0.09084600
P	1.51995100	-2.30812600	2.89697300
P	-0.03965800	0.37163900	4.43324300
P	2.78468700	0.89054500	2.49155400
P	-0.10719400	2.74958900	2.73964300
P	1.36507900	2.29119200	-0.36033700
P	0.03442400	-0.45047100	-2.01298700
P	0.09628200	-2.75191500	-0.21467400
P	-2.69941600	-0.92046500	0.04962800
P	-1.39648200	-2.40277000	2.87614100
P	-2.86360700	0.70344900	2.44913400
P	-1.50024900	2.19679600	-0.38273600
S	-2.85652400	5.59617300	1.05427100
C	-1.32021800	5.64253100	0.14272700
C	-1.31666400	5.62708100	-1.25531700
C	-0.10332700	5.65979400	0.83248700
C	1.11238500	5.69358600	0.14130700
C	1.10803400	5.67630900	-1.25635100

C	-0.10389800	5.60931700	-1.94380900
S	2.62728300	5.73530200	-2.18061800
S	-2.83786100	5.63377000	-2.17838500
S	-4.85155300	-3.99768700	-2.26031800
C	-3.45674200	-4.56717900	-1.31421200
C	-3.48966500	-4.57667100	0.08490900
C	-2.33231400	-5.04829000	-1.98509100
C	-1.26272100	-5.59934800	-1.27976300
C	-1.28146000	-5.59833000	0.11811500
C	-2.38771200	-5.06844800	0.79210100
S	0.11920300	-6.20758000	1.05245800
S	-4.93874800	-4.01633200	0.97302900
C	-3.80708200	4.40845100	0.11373500
C	-3.77737400	4.41661200	-1.28468100
C	-4.45903900	3.43200700	-1.99836100
C	-5.23395100	2.48166000	-1.33565300
C	-5.29215300	2.48296300	0.06305100
C	-4.55161100	3.42986500	0.77976500
S	-6.16184800	1.30368600	-2.29194600
C	-5.87218600	-0.18446500	-1.36078700
C	-5.97313900	-0.19662300	0.03546100
S	-6.32372300	1.30920800	0.92987100
C	-5.52110300	-1.34935800	-2.03898600
C	-5.30554500	-2.54062900	-1.34526800
C	-5.39714800	-2.55731600	0.04908100
C	-5.72972200	-1.38254400	0.73187200
S	2.65033400	5.70418900	1.05142500
C	1.50267800	-5.55894000	0.11941900

C	1.48430500	-5.56037700	-1.27841700
C	2.58755200	-4.98723900	0.79416300
C	3.66935700	-4.45224800	0.08745500
C	3.63666100	-4.44458800	-1.31186300
C	2.53285800	-4.96999300	-1.98324200
S	5.09331400	-3.83211900	0.97644400
C	5.49281300	-2.35613300	0.05229800
C	5.39746300	-2.34228400	-1.34191300
S	5.00558000	-3.81648400	-2.25787900
C	5.78485800	-1.17041800	0.73437400
C	5.98301200	0.02398900	0.03793300
C	5.87905100	0.03286100	-1.35786700
C	5.56756300	-1.14375000	-2.03546100
S	6.26680400	1.54298100	0.93417200
S	6.10922900	1.53065500	-2.28975600
C	5.13440800	2.67093400	-1.33449300
C	5.19329900	2.67649500	0.06414900
C	4.32371600	3.59032900	-1.99815400
C	3.60852400	4.55200500	-1.28584100
C	3.64119200	4.54857800	0.11230200
C	4.42055100	3.59838000	0.77956100
S	0.12098800	-6.26594100	-2.18020300
H	-0.10202500	5.62924300	1.91660800
H	-0.10299200	5.54072400	-3.02639200
H	-2.28944700	-4.99366200	-3.06765600
H	-2.38798500	-5.03247100	1.87648600
H	-4.37637500	3.39905100	-3.07939200
H	-5.72883500	-1.37417400	1.81718000

H	2.58404000	-4.94739900	1.87847100
H	5.78468400	-1.16221200	1.81969800
H	5.43113500	-1.12352300	-3.11136000
H	4.24181400	3.55254800	-3.07909800
H	-5.38747900	-1.32453200	-3.11513200
H	2.48782000	-4.91575300	-3.06572100
H	4.41300200	3.56124000	1.86400900
H	-4.54166800	3.39068000	1.86417200

References

[1] E. D. Glendening, C. R. Landis, and F. Weinhold, "Natural bond orbital methods," *Wiley interdisciplinary reviews: computational molecular science,* vol. 2, no. 1, pp. 1-42, 2012.

[2] H. Farrokhpour and H. Jouypazadeh, "Description of adenine and cytosine on Au (111) nano surface using different DFT functionals (PW91PW91, wB97XD, M06-2X, M06-L and CAM-B3LYP) in the framework of ONIOM scheme: Non-periodic calculations," *Chemical Physics,* vol. 488, pp. 1-10, 2017.

[3] F. Weigend and R. Ahlrichs, "Balanced basis sets of split valence, triple zeta valence and quadruple zeta valence quality for H to Rn: Design and assessment of accuracy," *Physical Chemistry Chemical Physics,* vol. 7, no. 18, pp. 3297-3305, 2005.
